# The effects of early mobilization in mechanically ventilated adult ICU patients: systematic review and meta-analysis

**DOI:** 10.3389/fmed.2023.1202754

**Published:** 2023-06-28

**Authors:** Lijie Wang, Yusi Hua, Luping Wang, Xia Zou, Yan Zhang, Xiaofeng Ou

**Affiliations:** ^1^Department of Critical Care Medicine, West China Hospital of Sichuan University, Chengdu, Sichuan, China; ^2^Department of Anesthesiology, West China Hospital of Sichuan University, Chengdu, Sichuan, China

**Keywords:** early mobilization, mechanical ventilation, ICU, mortality, ICU length of stay

## Abstract

**Background:**

The effects of early mobilization (EM) on intensive care unit (ICU) patients remain unclear. A meta-analysis of randomized controlled trials was performed to evaluate its effect in mechanically ventilated adult ICU patients.

**Methods:**

We searched randomized controlled trials (RCTs) published in Medline, Embase, and CENTRAL databases (from inception to November 2022). According to the difference in timing and type, the intervention group was defined as a systematic EM group, and comparator groups were divided into the late mobilization group and the standard EM group. The primary outcome was mortality. The secondary outcomes were ICU length of stay, duration of mechanical ventilation (MV), and adverse events. EM had no impact on 180-day mortality and hospital mortality between intervention groups and comparator groups (RR 1.09, 95% CI 0.89–1.33, *p* = 0.39). Systemic EM reduced the ICU length of stay (LOS) (MD −2.18, 95% CI −4.22–−0.13, *p* = 0.04) and the duration of MV (MD −2.27, 95% CI −3.99–−0.56, *p* = 0.009), but it may increase the incidence of adverse events in patients compared with the standard EM group (RR 1.99, 95% CI 1.25–3.16, *p* = 0.004).

**Conclusion:**

Systematic EM has no significant effect on short- or long-term mortality in mechanically ventilated adult ICU patients, but systematic EM could reduce the ICU LOS and duration of MV.

## 1. Introduction

Mechanically ventilated patients in ICUs are usually associated with short- or long-term complications, which are associated with increased mortality and mechanically ventilated duration, the longer length of ICU LOS and hospital LOS, reduced quality of life, and increased utilization of medical care (1). While the patients are being mechanically ventilated, EM has been proposed as a promising intervention to counteract these complications, and research suggests that it is a safe and feasible intervention (2, 3).

There was evidence of the feasibility of EM to strengthen muscles (4–6), improve Medical Research Council (MRC) and Barthel Index scores (7), and reduce the incidence of ICU-acquired weakness (8, 9), delirium rate (4, 10), and physical disability post–intensive care (11). It also prevented the occurrences of vein thrombosis, ventilator-associated pneumonia, and pressure sores (7, 12). Moreover, it shortens the duration of MV, length of ICU stay, and hospitalization (13, 14).

However, numerous studies found EM with no or inconclusive evidence for a benefit. Many meta-analyses have concluded that EM of ICU patients has no effects on improvements in the functional status, muscle strength, quality of life (QOL) or health care utilization outcomes, ICU LOS, hospital LOS, ICU mortality and hospital mortality (15, 16), and physical function- and mental health-related quality of life at 2–3 months and 6 months post-hospital discharge (12, 17). Most importantly, questions have recently arisen not only about the impact of EM on long-term outcomes but also about its safety. In an international, multicenter, randomized, controlled trial of 750 mechanically ventilated adult ICU patients, the TEAM study investigators and the ANZICS clinical trials group showed that an increase in EM did not improve survival, but it was associated with increased adverse events (18). On the other hand, because there is no unified concept of “early” in the EM literature, most studies believe that any mobilization activity is early if is commenced any time during the course of MV (19) or between 48 and 72 h after the start of MV (20, 21).

Therefore, based on a lack of consensus with published findings about the effects of EM in patients requiring MV in ICU, a meta-analysis of RCTs was conducted to comprehensively assess the benefits and adverse effects of EM in critically ill patients and requiring MV.

## 2. Methods

### 2.1. Protocol and registration

This study was based on the Preferred Reporting Items for Systematic Reviews and Meta-Analyses (PRISMA) guidelines (22). The protocol has been registered on the international prospective register of systematic reviews website (PROSPERO: https://www.crd.york.ac.uk/prospero/), and the registration number is CRD42022380303.

### 2.2. Eligibility criteria

Studies were included according to the following inclusion criteria: (1) Population: adult patients (≥18 years old) requiring MV at enrollment or during the ICU stay. (2) Design: RCT published in English. (3) Intervention: patients in the intervention group received systematic EM. Based on previously published meta-analyses (23), systematic EM was defined as any physical or occupational therapy targeting muscle activation, initiated within 3 days after ICU admission and performed according to a clearly defined protocol or specific clinical criteria in all eligible patients. (4) Comparators: patients in the control group received late mobilization (i.e., mobilization initiated 3 days or more after ICU admission) or standard EM (i.e., mobilization initiated within 3 days but less systematic) (23). (5) Outcomes: the primary outcome was mortality (including 180-day mortality and hospital mortality). The secondary outcomes were ICU LOS, duration of MV, and adverse events.

Studies that enrolled patients with pediatric, animal, or cell-based studies and studies published in narrative reviews, abstracts, commentaries, editorials case reports, and duplicate publications were excluded.

### 2.3. Information sources and search strategy

A computerized literature search was performed in Medline, Embase, and CENTRAL databases (from inception to November 2022) by two independent investigators using the keywords “intensive care unit,” “early mobilization,” “mechanical ventilation,” and “randomized controlled trial,” as well as their respective synonyms and derivations. The exact search strategy is provided in Supplementary File 1. The publication language was restricted to English.

After deduplication, two reviewers independently screened the titles and abstracts of all articles in order to detect the potential studies. Disagreements during the review process were resolved through discussion or consultation with an experienced senior reviewer. The pooled full-text references were then assessed to select eligible studies and when disagreement occurred, the dealing method is the same as mentioned above.

### 2.4. Data extraction

Two independent investigators adopted a standard collection form to extract related data from the included trials. The following information was extracted from each study: first author, year of publication, country, number of patients in intervention groups and comparator groups, patients' baseline characteristics, patient admission diagnoses, intervention description, time to first intervention, intervention frequency, intervention duration, and adverse events. Discrepancies between the researchers were resolved through discussion or arbitration by a third researcher.

### 2.5. Risk of bias assessment

The risk of bias in included studies was assessed using the Cochrane risk of bias tool, and the overall risk of bias for an individual trial was classified as high risk (when the risk of bias was high in at least one domain), low risk (when the risk of bias was low in all domains), or unclear (when the risk of bias was unclear in at least one domain) (24).

### 2.6. Data synthesis

Considering these studies differ in the timing and type of interventions, results are reported stratified by a comparator category (systematic EM, late mobilization, and standard EM). According to these studies (23, 25, 26), eligible comparators were categorized as: systematic EM (i.e., mobilization initiated within 3 days of admission to the ICU with), late mobilization (i.e., mobilization initiated 3 days or more after ICU admission), standard EM (i.e., mobilization initiated within 3 days but less systematically, or without clear initiated timing for mobilization). It is worth mentioning that one of these studies included an intervention description of the control group that received mobilization therapy after leaving the ICU (the ICU LOS is 18.3 ± 4.2 days), so this was defined as within the late mobilization category.

All statistical analyses were performed in this study using Review Manager 5.4 version (RevMan, The Cochrane Collaboration, Oxford, United Kingdom). For continuous variables (e.g., ICU LOS and duration of MV), mean differences (MDs) with 95% CIs were calculated using the inverse-variance (I-V) test, while for dichotomous variables (e.g., mortality and adverse events), risk ratios (RRs) with 95% confidence intervals (CIs) were calculated using the Mantel–Haenszel (M-H) test. In this study, some trials presented the indicators as a median and interquartile range (IQR), which were transferred into mean and standard deviation (SD) (27, 28). Comparable results were shown by fixed- or random effects and 95% confidence intervals.

Study heterogeneity was assessed by using the *I*^2^ statistics (29). If significant heterogeneity (*I*^2^ < 50%) was present, the fixed-effects model was used. Otherwise, the random-effects model was used. A two-sided *P*-value of ≤ 0.05 was considered to be statistically significant.

## 3. Results

### 3.1. Search results

**Figure 1:**
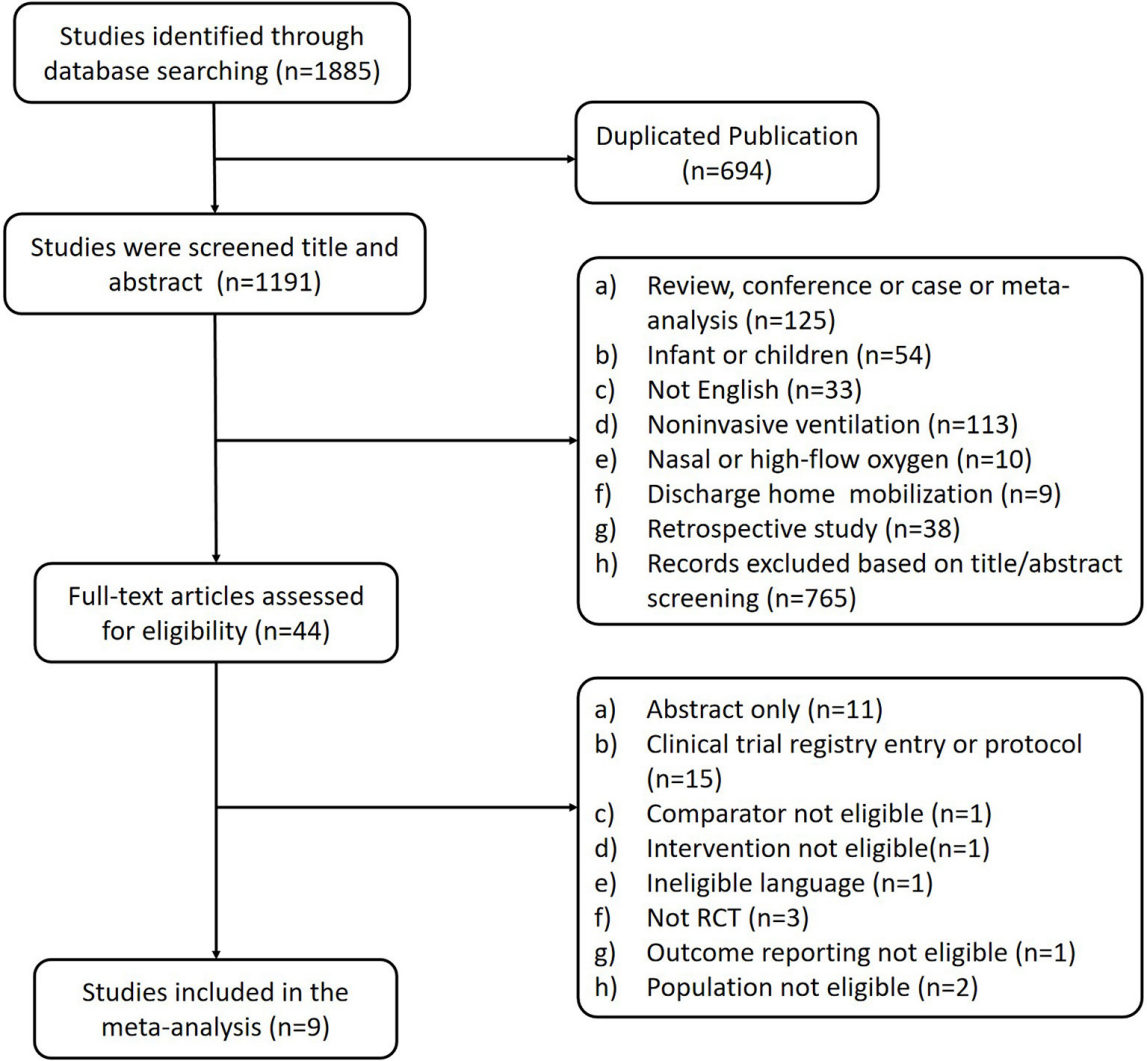
PRISMA flow diagram of the study selection.

Figure 1 shows the study selection process. The initial search identified 1,885 publications, of which 694 were excluded because of duplication. After reviewing the titles and abstracts, 1,147 were excluded because these articles' research type, population, or language were unqualified. After browsing full-text, nine RCTs (*n* = 1,756 patients) were eligible for inclusion and analysis in this meta-analysis (2, 4, 10, 13, 18, 30–33).

### 3.2. Study characteristics

The baseline characteristics of the included studies are presented in Table 1. Of the nine trials included, seven were published after 2015, while only two were published before that year, one in 2009 (2) and the other in 2014 (31). The included studies provided data from 883 people randomized to the intervention group and 873 people in the control group. In addition to the study conducted by Dong (30), the remaining eight trials reported the male-to-female ratio, which was about 42% in the intervention group and 43% in the comparator group. The mean age of the intervention group and the comparator group was similar, and the age difference in only two articles was large (4, 30), which may be due to the small sample size of the two trials and random error in sampling. Eight studies reported primary study outcomes [180-day mortality (18, 32, 33) and hospital mortality (2, 4, 10, 13, 31)], and these studies reported secondary outcome measures: ICU LOS (2, 4, 10, 13, 30–33), duration of MV (2, 4, 13, 30–32), and adverse events (2, 4, 10, 18, 30–33).

**Table 1:** Summary of included studies and study participants.

**Study**	**Country, timeframe**	**Population**	**Group**	**No. of participants**	**Females *n* (%)**	**Age Mean (SD)/ Median (IQR)**	**APACHEII score Mean (SD)/ Median (IQR)**	**Patient admission diagnoses**
Schweickert et al. (2)	USA, 2005–2007	Adult ICU patients, mechanically ventilated < 72 h, independent at baseline	Intervention	49	29 (59.2)	57.7 (36.3–69.1)	20.0 (15.8–24.0)	Lung injury (56%), COPD exacerbation (10%), acute exacerbation of asthma (9%), sepsis (15%), hemorrhage (3%), malignancy (3%), other (5%)
			Comparator	55	23 (41.8)	54.4 (46.5–66.4)	19.0 (13.3–23.0)	
Dong et al. (31)	China, 2010–2012	Adult ICU patients, mechanically ventilated between 48 and 72 h with expected ventilation of ≥ 1 week, clear consciousness, cardiovascular and respiratory stability	Intervention	30	9 (30.0)	55.3 (16.1)	15.0 (4.2)	Abdominal infections (18%), ARDS (32%), sepsis (7%), severe acute pancreatitis (15%), pneumonia (23%), COPD exacerbation (5%)
			Comparator	30	10 (33.3)	55.5 (16.2)	16.0 (4.1)	
Hodgson et al. (4)	Australia/New Zealand, 2013–2014	Adult ICU patients, mechanically ventilated within 72 h of ICU admission	Intervention	29	8 (25.9)	64 (12)	19.8 (9.8)	N/A
			Comparator	21	12 (57.1)	53 (15)	15.9 (6.9)	
Morris et al. (33)	USA, 2009–2014	Adult ICU patients, acute respiratory failure requiring mechanical ventilation	Intervention	150	84 (56.0)	55 (17)	NA	Acute respiratory failure (98%), coma (2%)
			Comparator	150	82 (54.7)	58 (14)	NA	
Schaller et al. (10)	USA/Germany, 2011–2015	Adult surgical ICU patients, mechanically ventilated for < 48 h and for at least further 24 h, functionally independent at baseline	Intervention	104	39 (37.5)	66 (48–73)	16 (12–22)	Visceral surgery (27%), vascular surgery (17%), ENT and ophthalmological surgery (10%), transplant surgery (4%), neurosurgery (3%), orthopedic surgery (3%), thoracic surgery (3%), gynecological surgery (2%), urological surgery (1%), plastic surgery (1%), medical or neurological diagnosis (6%), trauma (26%)
			Comparator	96	35 (36.5)	64 (45–76)	17 (11–22)	
Dong et al. (13)	China, 2012–2015	Adult patients, prolonged mechanical ventilation > 72 h, eligible for coronary artery bypass surgery	Intervention	53	33 (62.3)	62.6 (12.8)	16.3 (4.2)	Coronary artery bypass surgery (100%)
			Comparator	53	31 (58.5)	60.2 (15.1)	17.2 (4.3)	
Eggmann et al. (32)	Switzerland, 2012–2016	Adult ICU patients, expected to stay on mechanical ventilation for at least 72 h, independent before critical illness	Intervention	58	22 (37.9)	65 (15)	23.0 (7.0)	Cardiac surgery (18%), neurology/neurosurgery (8%), other surgery (12%), gastroenterology (12%), trauma (4%), respiratory insufficiency (22%), hemodynamic insufficiency (23%), other (2%)
Dong et al. (30)	China, 2019–2020	Adult ICU patients, Prolonged MV (> 72 h), clear consciousness, cardiovascular and respiratory stability, no history of chronic mental illness or COPD	Intervention	39	NA	59.05 ± 17.61	15.90 ± 6.01	N/A
			Comparator	41	NA	64.44 ± 14.72	17.78 ± 8.40	
Hodgson et al. (18)	Australia/ New Zealand/ Germany/ Ireland/UK/ Brazil, 2018–2021	Adult ICU patients (≥ 18 years of age), mechanical ventilation, condition was sufficiently stable to make mobilization potentially possible	Intervention	371	128 (34.5)	60.5 ± 14.8	18.2 ± 6.8	Sepsis (66.3%), Trauma (3.9%), COVID-19 (2.3%), others (27.5%)
			Comparator	370	146 (39.5)	59.5 ± 15.2	18 ± 6.9	

Considering the difference between studies in the timing and type of interventions, some results are reported stratified by a comparator category (systematic EM, late mobilization, and standard EM). According to the study definition, five studies were classified (10, 18, 30–32) as comparing systematic EM vs. standard EM, and the other four studies (2, 4, 13, 33) were classified as systematic EM vs. late mobilization (in Table 2). Different studies intervene in different ways, including head up, transferring from supination to sitting, standing, and walking, and other goal-oriented mobilization protocols. The frequency of intervention was once daily, twice daily, or three times daily. The duration of the intervention ranged from 20 to 60 min in the intervention group and from 0 to 0.2 h in the control group (in Table 2).

**Table 2:** Details on study interventions and comparators.

**Study**	**Group**	**Intervention description**	**Time to first intervention Median (IQR) (days)**	**Intervention frequency**	**Intervention duration Mean (SD)/ Median (IQR)**
**Systematic early mobilization vs. Standard early mobilization**					
Dong et al. (31)	Intervention	Heading up actively, transferring from the supine position to sitting position, sitting at the edge of the bed, sitting in chair, transferring from sitting to standing, ambulating bedside and changed every 2 h	N/A	Twice daily	According to the condition of patients
	Comparator	Not described	N/A	N/A	N/A
Schaller et al. (10)	Intervention	Early, goal-directed mobilization algorithm: the goal for a specific day was set either to level 0 (no mobilization), level 1 (passive range of motion exercises in the bed), level 2 (sitting), level 3 (standing), or level 4 ambulation)	N/A	Once daily	Depending on the condition of patients
	Comparator	Standard care except for early, goal directed mobilization	N/A	Once daily	N/A
Eggmann et al. (32)	Intervention	Motor-assisted bed-cycle, standard exercises for both upper and lower limbs exercises for both upper and lower limbs, in-bed exercise, sitting, standing and walking	2.0 (1.4–2.8) after ICU admission	maximum 3 times daily, 7 days per week	25 min (19.5–27)
	Comparator	European standard Physiotherapy including early mobilization, respiratory therapy and passive or active exercises	2.0 (1.4–2.8) after ICU admission	Once daily	18 min (14–21)
Dong et al. (30)	Intervention	Rehabilitation therapy consisted of six levels: level 0, turning over; level 1–2, sit up; level 3, sitting on the edge of bed; level 4, standing up or sitting in a chair; level 5, moved from the bed and walked	N/A	N/A	Tailored depending on the condition of patients
	Comparator	Standard care	N/A	N/A	N/A
Hodgson et al. (18)	Intervention	Senior physiotherapists led the intervention and participated in interdisciplinary discussions and reviews of a safety checklist	N/A	Once daily	20.8 ± 14.6 min
	Comparator	The level of mobilization that was normally provided in each ICU	N/A	Once daily	8.8 ± 9.0 min
**Systematic early mobilization vs. Late mobilization**					
Schweickert et al. (2)	Intervention	Passive range of motion, active-assisted and active-independent exercises, bed mobility exercises, Sitting balance activities, transfer training, pre-gait exercises and walking	1.5 (1.0–2.1) after intubation	Once daily	0.3 h (0.2–0.5) per day during ventilation 0.2 h (0.1–0.3) per day without ventilation
	Comparator	standard care with physical and occupational therapy delivered as ordered by the primary care team	7.4 (6.0–10.9) after intubation	N/A	0.0 h (0.0–0.0) per day during ventilation 0.2 h (0.0–0.4) per day without ventilation
Morris et al. (33)	Intervention	Passive range of motion, physical therapy and progressive resistance exercise	1 (0–2) after ICU admission	3 times daily, 7 days a week	N/A
	Comparator	Weekday physical therapy when ordered by the clinical team	7 (4–10) after ICU admission	5 days a week	N/A
Dong et al. (13)	Intervention	head up, transferring from supination to sitting, sitting on the edge of bed, sitting in a chair, transferring from sitting to standing, and walking along a bed	N/A	Twice daily	N/A
	Comparator	Received rehabilitation therapy with the help of family after leaving the ICU	N/A	N/A	N/A
Hodgson et al. (4)	Intervention	Functional activities comprising walking, standing, balance exercises, sitting in or out of bed, sitting and rolling (the patient could receive assistance from staff or equipment but the patient actively participated in the exercise at the highest functional level)	3 (2–4) after ICU admission	Once daily	About 30–60 min depending on the condition of patients
	Comparator	Passive movements (the same mobilization equipment was available in both the control group and the intervention group)	4 (3–5) after ICU admission	Once daily	About 5–10 min per day

### 3.3. Risk of bias assessment

**Figure 2:**
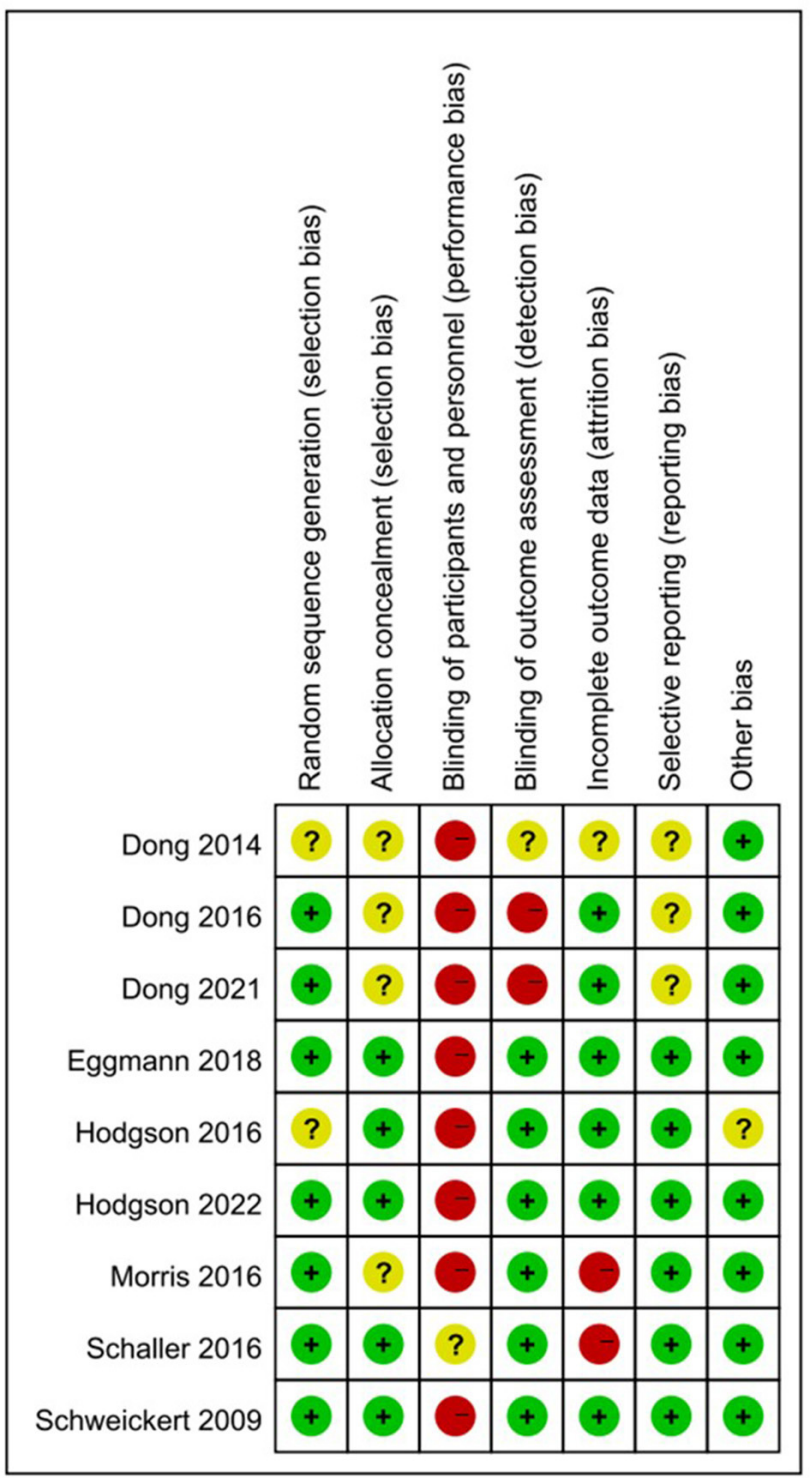
Risk of bias assessment for the included studies.

The details of the risk of bias assessment are summarized in Figure 2. Seven studies (78%) were at low risk of bias of the random sequence generation. A suitable method of allocation concealment was used in five studies (56%). Because the patients in the intervention group needs to rehabilitate, blinding of participants and personnel was not possible, and eight studies (89%) were at high risk of bias. Six studies (67%) reported blinding of the outcome assessment. Incomplete outcome data may exist in two studies (22%), and three studies (33%) could be reporting selective.

### 3.4. Mortality

**Figure 3:**
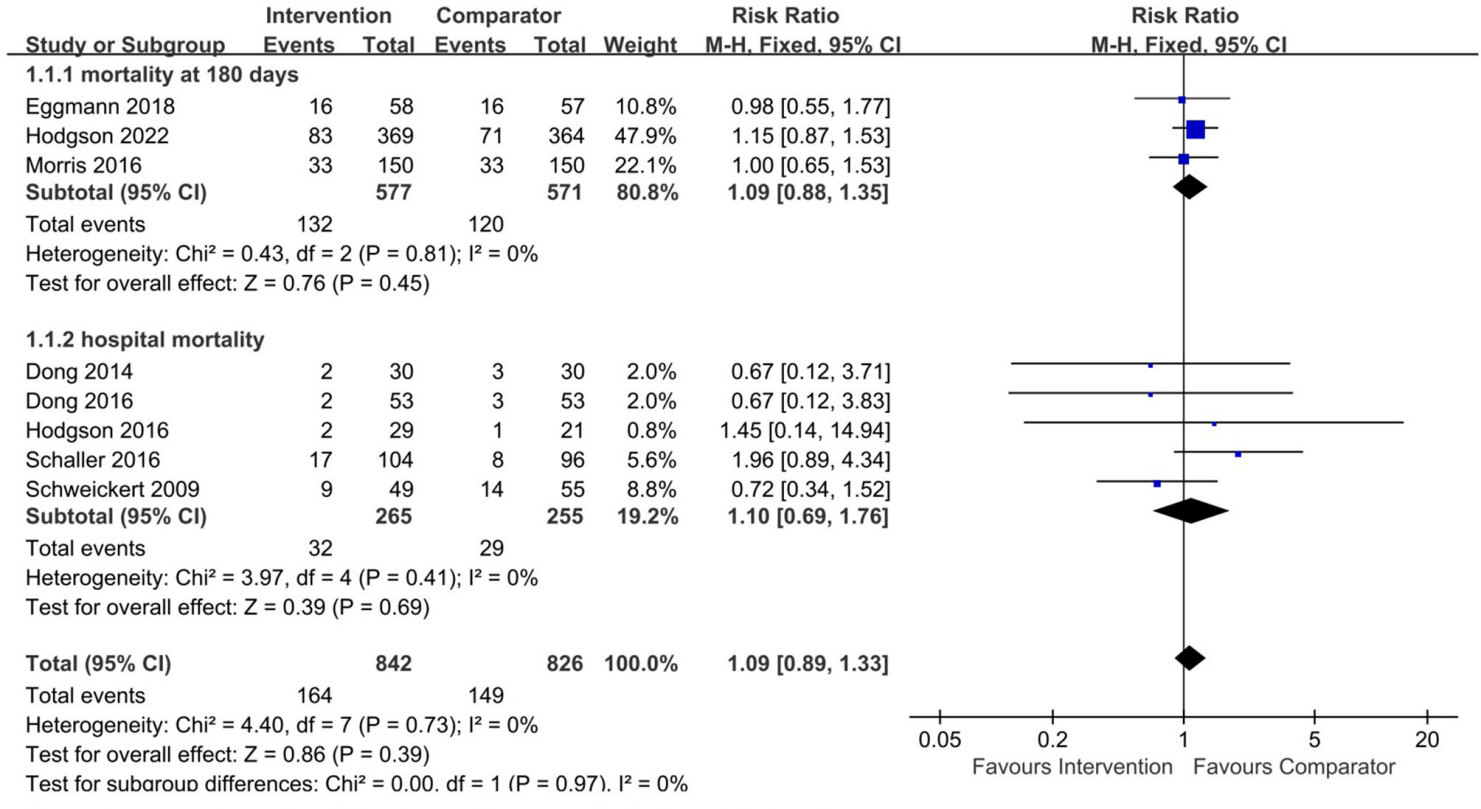
Forest plot for early mobilization on effect mortality in the included studies. Intervention group = systematic early mobilization group, Comparator = late mobilization or standard early mobilization.

As shown in Figure 3, eight studies reported mortality at different time points. Among them, three studies (18, 32, 33) reported 180-day mortality that included 577 patients in the intervention group (the systematic EM group) and 571 patients in the comparator group (the late mobilization group and the standard EM group), and there was no significant difference in 180-day mortality between the two groups (RR 1.09, 95% CI 0.88–1.35, *I*^2^ = 0%, test for overall effect: Z = 0.76, *p* = 0.45). As for hospital mortality, there were five studies included in this analysis with 520 patients (2, 4, 10, 13, 31), and no significant difference was found in mortality between the two groups (RR 1.10, 95% CI 0.69–1.76, *I*^2^ = 0%, test for overall effect: Z = 0.39, *p* = 0.69). The results of subgroup analysis showed no difference in mortality between the systematic EM group and standard EM or the late mobilization group at any time points (RR 1.09, 95% CI 0.89–1.33, *I*^2^ = 0%, test for overall effect: *Z* = 0.86, *p* = 0.39).

### 3.5. ICU length of stay

**Figure 4:**
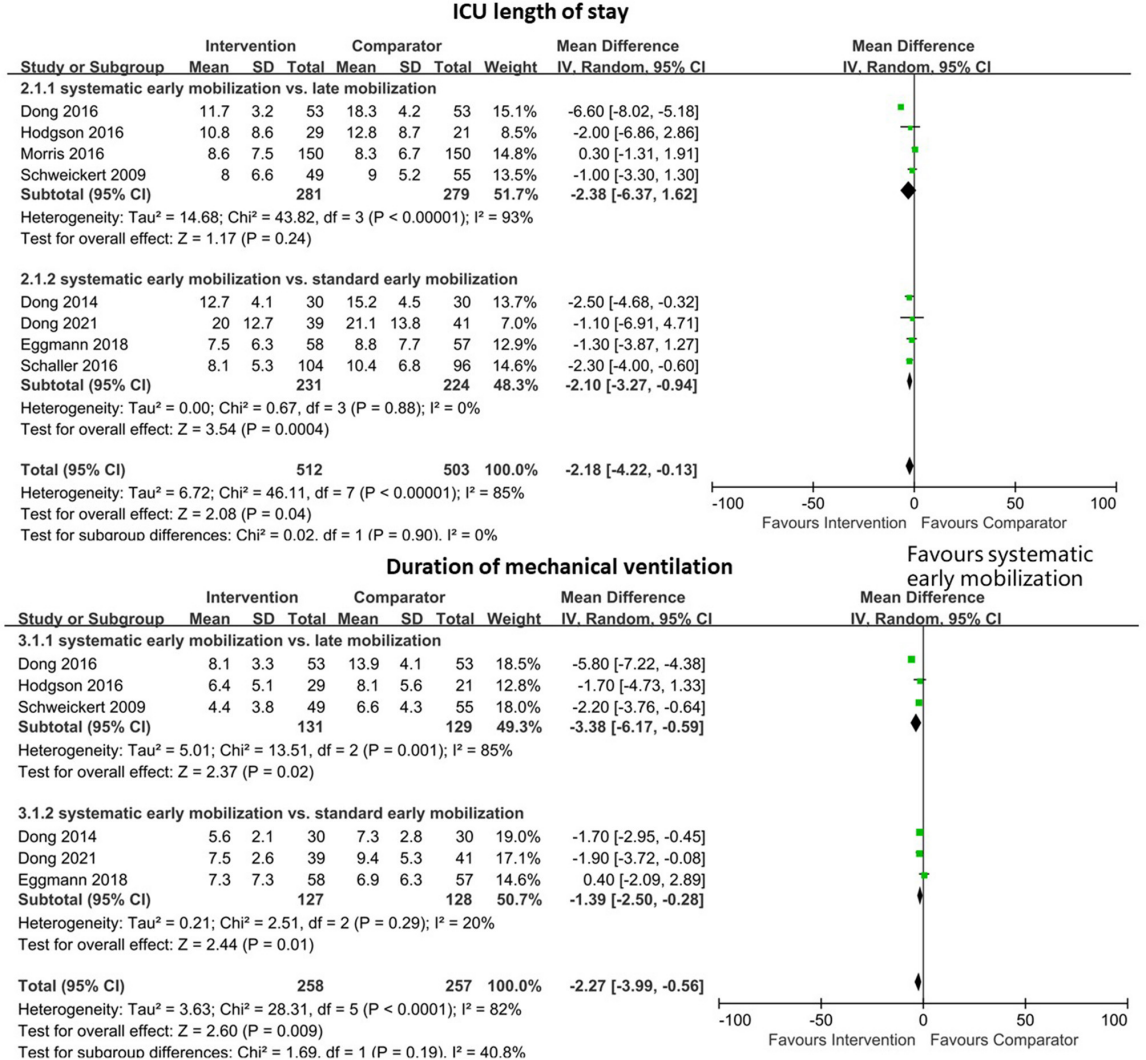
Forest plot for early mobilization effect on ICU length of stay in the included studies.

Eight studies reported the relationship between EM and ICU LOS. In the subgroup analysis, there are four studies that adopted systematic EM and late mobilization (2, 4, 13, 33), and no significant difference was found between these two groups (MD −2.38, 95% CI −6.37–1.62, *I*^2^ = 93%, test for overall effect: Z = 1.17, *p* = 0.24). In addition, the other four studies (10, 30–32) had an impact on systemic EM and standard EM for LOS in ICU. Compared with the standard EM group, there was a statistically significant reduction of ICU LOS in the systematic EM group (MD −2.10, 95% CI −3.27–−0.94, *I*^2^ = 0%, test for overall effect: Z = 3.54, *p* < 0.001). A pooled analysis of these studies showed a significant mean difference and favored the systematic EM group (MD −2.18, 95% CI −4.22–−0.13, *I*^2^ = 85%, test for overall effect: Z = 2.08, *p* = 0.04, n = 1,015) (Figure 4).

### 3.6. Duration of mechanical ventilation

Six studies analyzed 515 patients who reported the duration of MV. The pooled analysis of the data indicated a decreased trend in the duration of MV following systematic EM (MD −2.27, 95% CI −3.99–−0.56, *I*^2^ = 82%, test for overall effect: Z = 2.60, *p* = 0.009) (Figure 4). In the subgroup analysis, there was a statistically significant mechanically ventilated duration in the systematic EM group, compared with the late mobilization group (2, 4, 13) and the standard EM group (30–32) (MD −3.38, 95% CI −6.17–−0.59, *I*^2^ = 85%, test for overall effect: Z = 2.37, *p* = 0.02 and MD −1.39, 95% CI −2.50–−0.28, *I*^2^ = 20%, test for overall effect: Z = 2.44, *p* = 0.01, respectively) (Figure 4).

### 3.7. Adverse events

**Figure 5:**
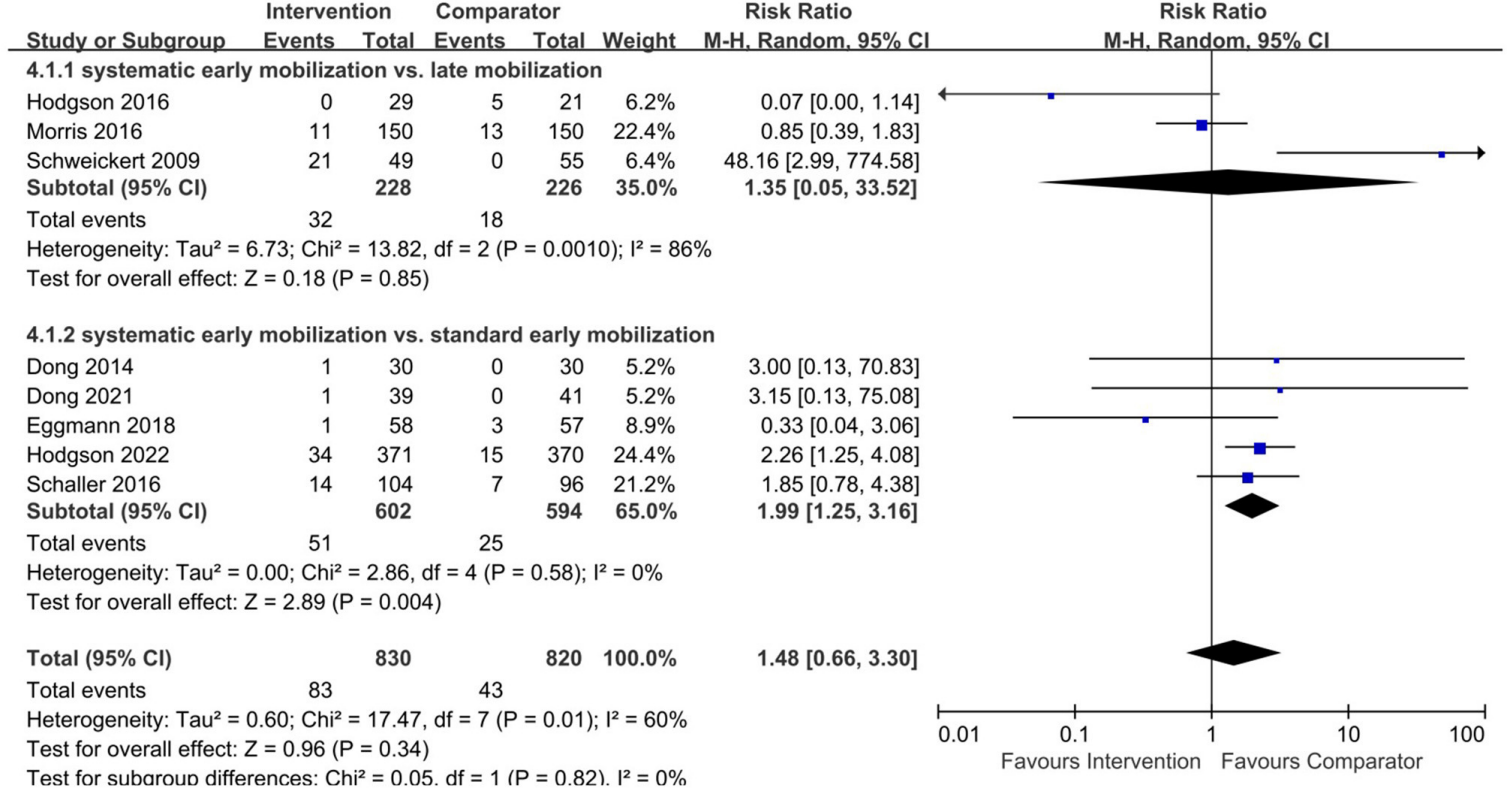
Forest plot for early mobilization effect on adverse events in the included studies.

A total of eight trials with 1,650 patients reported different adverse events among participants. These trials reported adverse events including decreased desaturation, agitation, dislodgement of arterial line or nasogastric tube, dyspnea, dizziness, cardiac arrhythmia, altered blood pressure, and cerebrovascular accident (2, 4, 10, 18, 30–33). The adverse events were not significantly different between the systemic EM group and the late mobilization group (RR 1.35, 95% CI 0.05–33.52, *I*^2^ = 86%, test for overall effect: Z = 0.18, *p* = 0.85) (2, 4, 33). However, there were more adverse events in the systemic EM group compared to the standard EM group (RR 1.99, 95% CI 1.25–3.16, *I*^2^ = 0%, test for overall effect: Z = 2.89, *p* = 0.004) (10, 18, 30–32) (Figure 5).

## 4. Discussion

This meta-analysis included 9 RCTs, and it was found that systematic EM had no effect on short- or long-term mortality in mechanically ventilated adult ICU patients, but it could reduce the LOS in ICU and the duration of MV. While systemic EM may increase the incidence of adverse events in patients compared with standard EM.

The meta-analysis found that EM in the ICU had no effects on 180 days mortality and hospital mortality. There have been many studies on the impact of EM on mortality in ICU patients. After comparing the effects of EM and late mobilization, standard EM or no mobilization, Dominik (23) argued that none of them had any effect on patients' short-term mortality (hospital mortality) and long-term mortality (6-month mortality), which the current study findings support. A systematic review (34) about the impact of mobilization on the mortality of ICU patients demonstrated that mobilization had no positive effects on short- or long-term mortality. The results were consistent with the present study findings, but the current meta-analysis was the inclusion of a multicenter, high-quality, large-population RCTS study published in the New England Journal in October 2022 (18), which added strong evidence to the results.

This study showed that both ICU LOS and the duration of the mechanical ventilator were approximately reduced by 2 days in the EM group. The included four studies (10, 30–32) comparing the length of ICU stay between systematic EM and standard EM showed little heterogeneity and a significant difference between the two groups, suggesting systematic EM within 3 days of ICU admission can effectively reduce the length of ICU stay. Similar results have been found in other systematic reviews. Klem et al. (35) suggested that EM can shorten ICU stay by 1 day but has no effect on the total hospital LOS and also about the effects of systematic EM on the duration of the mechanical ventilator. Zhang et al. (36) reported the same positive results in a systematic review. Monsees et al. (37) also implied the same trend toward a reduction in the duration of mechanical ventilators with EM. It is thought that EM can reduce ICU-acquired weakness (38), which may associate with a prolonged duration of mechanical ventilator (39, 40).

In terms of safety and adverse events, there were eight trials that reported adverse events. Hodgson (18) reported 34 patients with adverse events in the EM group and 15 patients with adverse events in the usual care group, suggesting that the incidence of adverse events in the EM group was higher than that in the usual care group (*P* = 0.005). While in the other studies, there was no difference in the incidence of adverse events between the intervention group and the comparator group. Although serious adverse events were very rare, they still occurred. For example, Schweickert (38) reported a case of desaturation of < 80%. Therefore, it is believed that the initiation of EM should be very cautious.

There are some limitations in this study. First, some of the included studies had small sample sizes. In three studies (4, 30, 31), there were < 100 total participants, which is more likely to overestimate the effects. Second, our conclusions may be limited by the poor quality and bias of some of the studies. The performance bias and detection bias in these two articles are high-risk, and selection bias and reporting bias are unclear (13, 30). Third, the definition of EM is not clear in those included studies. Some studies suggest that it should be limited to 3 days (25, 26), while others suggest that it should be limited to 7 days (23). Different definitions may lead to different subgroups, which may affect results. In addition, some other factors cannot be ignored, such as the mode and duration of mobilization treatment, which vary greatly between studies. The lack of detailed information may affect the accuracy of this study.

## 5. Conclusion

Although EM does not improve short- or long-term mortality in mechanically ventilated adult ICU patients, this systematic review found that systematic EM could reduce the ICU LOS and duration of MV, but it may increase the incidence of adverse events compared with standard EM, which suggest that EM should be initiated carefully. However, given the potential limitations of this study and the substantial heterogeneity among the included trials, the results of this study should be interpreted with caution. Further large-scale and well-conducted RCTs are needed to validate our current findings.

## Author contributions

XO: conceptualization and supervision. LiW: methodology and writing—review and editing. LiW and YH: formal analysis. LiW, YH, XZ, YZ, and LuW: data curation. LiW and LuW: writing—original draft preparation. All authors have read and agreed to the published version of the manuscript.
