# Supplementary material for: The effects of early mobilization in mechanically ventilated adult ICU patients: systematic review and meta-analysis
Source: Front Med (Lausanne). 2023 Jun 28;10:1202754. doi: 10.3389/fmed.2023.1202754 (PMC10336545; doi:10.3389/fmed.2023.1202754)
Supplement: Supplementary file 1 [file Data_Sheet_1.docx]

**Search Strategies**

1. Critical Illness
2. Critically Ill
3. Critical patient
4. Intensive Care Unit
5. intensive care
6. ICU
7. critical care
8. Mechanical Ventilation
9. ventilation support
10. Artificial Respiration
11. Early Ambulation
12. Accelerated Ambulation
13. Early Mobilization
14. Early Mobilisation
15. early mobility
16. Mobilization
17. Mobilisation
18. Ambulation
19. Motion
20. Mobility
21. Exercise Therapy
22. Exercise Therapies
23. Rehabilitation Exercises
24. Remedial Exercise
25. Exercise
26. occupational therapy
27. Occupational Therapies
28. Physical Fitness
29. cycle ergometry
30. transfers out of bed
31. transfer training
32. walking
33. PICS
34. ICUAW
35. Rehabilitation
36. physical therapy
37. physiotherapy
38. muscle training
39. diary
40. randomized controlled trial
41. controlled clinical trial
42. clinical trial
43. randomized
44. randomised
45. randomly
46. trial
47. placebo
48. controlled clinical

*EBM Reviews - Cochrane Central Register of Controlled Trials <October 2022>*

1. (Critical Illness or Critically Ill or Critical patient or Intensive Care Unit or intensive care or ICU or critical care).af.
2. (Mechanical Ventilation or ventilation support or Artificial Respiration).af.
3. (Early Ambulation or Accelerated Ambulation or Early Mobilization or Early Mobilisation or early mobility or Mobilization or Mobilisation or Ambulation or Motion or Mobility or Exercise Therapy or Exercise Therapies or Rehabilitation Exercises or Remedial Exercise or Exercise or occupational therapy or Occupational Therapies or Physical Fitness or cycle ergometry or cycle or bicycle or transfers out of bed or transfer training or walking or PICS or ICUAW or ICU-AW or Rehabilitation or physical therapy or physiotherapy or muscle training or diary).af.
4. (randomized controlled trial or controlled clinical trial or clinical trial or randomized or randomised or randomly or trial or placebo or controlled clinical).ti,ab. not (animals not humans).sh.
5. 1 and 2 and 3 and 4

*Embase <1974 to 2022 November 17>*

1. (Critical Illness or Critically Ill or Critical patient or Intensive Care Unit or intensive care or ICU or critical care).af.
2. (Mechanical Ventilation or ventilation support or Artificial Respiration).af.
3. (Early Ambulation or Accelerated Ambulation or Early Mobilization or Early Mobilisation or early mobility or Mobilization or Mobilisation or Ambulation or Motion or Mobility or Exercise Therapy or Exercise Therapies or Rehabilitation Exercises or Remedial Exercise or Exercise or occupational therapy or Occupational Therapies or Physical Fitness or cycle ergometry or cycle or bicycle or transfers out of bed or transfer training or walking or PICS or ICUAW or ICU-AW or Rehabilitation or physical therapy or physiotherapy or muscle training or diary).af.
4. (randomized controlled trial or controlled clinical trial or clinical trial or randomized or randomised or randomly or trial or placebo or controlled clinical).ti,ab. not (animals not humans).sh.
5. 1 and 2 and 3 and 4

*Ovid MEDLINE(R) and Epub Ahead of Print, In-Process, In-Data-Review & Other Non-Indexed Citations, Daily and Versions <1946 to November 17, 2022>*

1. (Critical Illness or Critically Ill or Critical patient or Intensive Care Unit or intensive care or ICU or critical care).af.
2. (Mechanical Ventilation or ventilation support or Artificial Respiration).af. 57496
3. (Early Ambulation or Accelerated Ambulation or Early Mobilization or Early Mobilisation or early mobility or Mobilization or Mobilisation or Ambulation or Motion or Mobility or Exercise Therapy or Exercise Therapies or Rehabilitation Exercises or Remedial Exercise or Exercise or occupational therapy or Occupational Therapies or Physical Fitness or cycle ergometry or cycle or bicycle or transfers out of bed or transfer training or walking or PICS or ICUAW or ICU-AW or Rehabilitation or physical therapy or physiotherapy or muscle training or diary).af.
4. (randomized controlled trial or controlled clinical trial or clinical trial or randomized or randomised or randomly or trial or placebo or controlled clinical).ti,ab. not (animals not humans).sh.
5. 1 and 2 and 3 and 4
